# Foreign Bogus Diploma Mills

**Published:** 1883-06

**Authors:** 


### FOREIGN BOGUS DIPLOMA MILLS.

An extensive trade in bogus decorations has recently been discovered in Naples, Italy. For a number of years there have existed several fraudulent committees who issued, for a round price, orders, academic degrees, prize medals and other decorations of a similar nature. Lately one of the committees had the impudence to make a decoration offer to the King of Bavaria, with a promise to put his name into the "libro d' oro" of Naples, which in reality has no existence. This brought about the explosion. Among a very extended correspondence no less than 700 bogus diplomas were found, and many requests from even well known persons. If the goverment of Naples was less discreet, many a decoration-and-diploma-maniac would have cause to blush. In connection with this Dr. L. Ribolla Nicodemi, of Palermo, publishes the following letter: "There is an 'Academy of Arts and Sciences' in Naples, which issues titles and diplomas for a certain sum of money, after the pattern of the 'Reale Associazione de' Benemeriti Italian,' in Palermo, of the 'Circolo promotore Partenopeo' in Naples, and others. There has been no exhibition lately in Naples, but a permanent exhibition of the 'Giambattista Vigo' which sells its 'gold medals' for about four dollars apiece."—*Zahntechnische Reform.*

